# The relevance of patient-reported outcome measures in hypopituitarism: HIV therapy adherence and suicidal ideations resolved by GH replacement

**DOI:** 10.1530/EDM-25-0047

**Published:** 2025-11-03

**Authors:** Linus Hesse, Loreen Richter, Lukas Maurer, Joachim Spranger, Knut Mai, Christian J Strasburger, Linus Haberbosch

**Affiliations:** ^1^ Charité – Universitätsmedizin Berlin, Corporate Member of Freie Universität Berlin, Humboldt-Universität zu Berlin, Department of Endocrinology and Metabolism; European Reference Network On Rare Endocrine Diseases (ENDO-ERN), Berlin, Germany; ^2^ Pituitary Tumor Center of Excellence at Charité – Universitätsmedizin Berlin, Corporate Member of Freie Universität Berlin, Humboldt-Universität zu Berlin, Berlin, Germany; ^3^ Cambridge Endocrine Molecular Imaging Group, Institute of Metabolic Science, University of Cambridge, National Institute for Health Research Cambridge Biomedical Research Centre, Addenbrooke’s Hospital, Cambridge, UK; ^4^ Charité – Universitätsmedizin Berlin, Corporate Member of Freie Universität Berlin, Humboldt-Universität zu Berlin, Department of Neurosurgery, Berlin, Germany; ^5^ Department of Human Nutrition, German Institute of Human Nutrition Potsdam-Rehbrücke, Nuthetal, Germany

**Keywords:** growth hormone, quality of life, HIV, hypopituitarism, PROMs

## Abstract

**Summary:**

Extensive research has examined the effects of growth hormone (GH) replacement therapy for patients with hypopituitarism and GH deficiency. Positive outcomes, encompassing both conventional clinical markers and improvements in quality of life (QoL), have led to its incorporation in current clinical practice guidelines. Herein, we present how routine use of the patient-reported outcome measures information system (PROMIS) as an intuitive tool portraying patient-reported outcome measures (PROMs) can help guide and follow-up therapeutic decisions and bridge the gap between clinical parameters and patient experiences. This approach is illustrated by a patient with panhypopituitarism and HIV with low antiretroviral medication adherence. Following the initiation of GH replacement therapy, PROMIS assessments documented QoL improvements that enabled full resumption of antiretroviral medication, accompanied by resolution of previously experienced adverse effects.

**Learning points:**

- GH replacement can be an important component of hormone replacement therapy in individuals with hypopituitarism and suicidal ideations.
- Incorporating PROMs into endocrinological care can help guide therapy decisions in patients with hypopituitarism.
- Clinical signs attributed to side effects of antiretroviral therapy may overlap with those of GH deficiency, and GH replacement therapy can potentially increase medication adherence.

## Background

Despite established diagnostic protocols and extensive evidence and treatment guidelines supporting the safety and efficacy of growth hormone (GH) replacement in adults (1), variability in clinical practice persists, sometimes resulting in delayed or withheld therapy for patients who could substantially benefit from this replacement therapy. GH replacement in adults is recognized for its ability to optimize body composition, preserve skeletal mass, normalize cardiovascular risk factors and, consequently, improve both physical and psychological well-being (1). Moreover, sustained improvements in quality of life (QoL) have been documented across different disease-specific and generic psychometric instruments (2, 3, 4). In HIV-positive patients, GH secretion is impaired in approximately 30%; however, the optimal treatment strategy is uncertain (5).

This case report highlights the potential of using validated, cross-disease comparable Patient-Reported Outcome Measures (PROMs) such as the Patient Reported Outcome Measure Information System (PROMIS)-33 in clinical routine and demonstrates the impact of GH replacement on a patient suffering from panhypopituitarism, HIV infection, and poorly tolerated antiretroviral therapy marked by low adherence and depression.

## Case presentation

A 60-year-old male patient was referred to our department after resolving an adrenal crisis. His medical history included an empty sella and panhypopituitarism after a surgical exploration of the sella 20 years ago, which revealed lymphocytic and monocytic infiltration after traversed sinusitis. The patient was under regular doses of hydrocortisone (30 mg daily), thyroxin (100 μg daily), and testosterone (81 mg daily, transdermal). GH supplementation, however, had been deemed unnecessary by his previous endocrinologist. The former triathlete reported severe muscle fatigue, weakness, and exhaustion, which persisted after stabilization of the adrenal crisis and subsequent oral hydrocortisone treatment.

Since the diagnosis of an HIV infection 35 years prior, the patient was suffering from depression, which he reported had significantly worsened after the exploration of the sella.

Throughout his HIV treatment, the patient experienced severe adverse reactions to multiple antiretroviral regimens. He discontinued initial Azidothymidine (AZT) therapy several times. Two years after surgical exploration of the sella, AZT was replaced by Emtricitabine, Tenofovir, and Abacavir. Later, Abacavir had been switched to Bictegravir due to persistent side effects. However, he continued to suffer from chronic diarrhea, severe muscle pain, and weakness, which resulted in a significant decline in QoL for the former athlete. Recurrent interruptions of the HIV therapy resulted in direct consequences, including an HIV-associated zoster oticus with tinnitus and facial nerve palsy in 2017.

Not able to tolerate his medication and having given up on life, he applied for hospice care. As he wanted to ‘die in Berlin’, he moved to the Berlin apartment of a long-time friend. Here, he collapsed and was admitted to a nearby hospital that resolved an adrenal crisis before being referred to our center.

## Investigation

**Figure 1:**
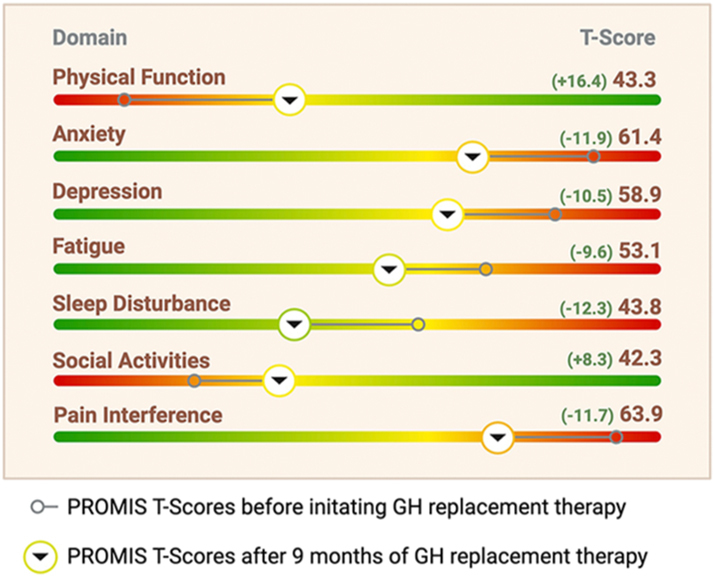
Example of PROM feedback for the treating physicians. T-scores (10 = 1 standard deviation, 50 = median) are shown on color gradient bars ranging from 20 to 80, with green indicating better outcomes and red indicating worse outcomes.

After resolving the adrenal crisis and tapering the hydrocortisone dose to 30 mg daily, we assessed the endocrinological status, revealing adequate supplementation of the thyrotropic and gonadal axis. We furthermore confirmed the concomitant somatotropic insufficiency (IGF-1-SDS: −4.60, GH after GHRH/arginine stimulation test: <0.5 ng/mL). QoL was assessed via an app-based German version of PROMIS and is represented in Fig. 1. Our patient’s T-Scores (10 = 1 standard deviation, 50 = median) were markedly elevated compared to the age- and sex-matched German general population (presented as median (10%-90% confidence interval)) (6) in the domains of pain interference (75.6; reference: 52.6 (37.7–64)), fatigue (62.7; ref: 46.9 (32.5–59.1)), anxiety (73.3; ref: 50.7 (38.3–60.8)), sleep disturbance (56.6; ref: 47.8 (36.5–59.9)), and depression (69.4; ref: 50.7 (37.3–61.1)), while also reduced T-Scores (indicating worse findings) in the domains of physical function (26.9; ref: 50.4 (38.5–63.1)) and social activities (34.0; ref: 51.5 (39.8–64.7)).

## Outcome and follow-up

We started the patient on a daily dose of 0.2 mg of recombinant human GH in addition to the continuation of the previous replacement therapies for the other pituitary axes. The patient reported noticeable improvements in physical endurance and overall well-being, which improved further after increasing the dose to 0.4 mg. He was able to resume his antiretroviral therapy with Emtricitabine, Tenofovir, and Bictegravir. The sole remaining side effect he experienced was muscle aches. After increasing GH to 0.6 mg (IGF-1-SDS: −0.89), he reported no relevant side effects of the antiretroviral therapy anymore.

A second PROMIS assessment showed major improvements in all affected domains: pain interference decreased from 75.6 to 63.9 (ref: 52.6 (37.7–64)), fatigue from 62.7 to 53.1 (ref: 46.9 (32.5–59.1)), anxiety from 73.3 to 61.4 (ref: 50.7 (38.3–60.8)), sleep disturbance from 56.6 to 43.8 (ref: 47.8 (36.5–59.9)), and depression from 69.4 to 58.9 (ref: 50.7 (37.3–61.1)), while also improved physical function from 26.9 to 43.3 (ref: 50.4 (38.5–63.1)) and social activities scores from 34.0 to 42.3 (ref: 51.5 (39.8–64.7)) (represented in Fig. 1). He was able to resume physical activities and fully distanced himself from any suicidal ideations. His application for hospice care was withdrawn.

## Discussion

We present a case that highlights the importance of PROMs and GH replacement for the QoL of patients with complete anterior pituitary failure and multiple somatic and psychosomatic challenges.

Despite successful treatment of initial pituitary disease, QoL of individuals with residual hormonal deficits often remains impaired, with reduced physical and social functioning, emotional instability, and increased pain sensitivity, among others (4, 7).

In this patient, the already reduced QoL likely exacerbated the psychological impact that adverse effects of his antiretroviral therapy exerted on him, which led to discontinuation of the HIV therapy. Notably, symptoms (weakness, muscle pain, and chronic diarrhea) attributed to adverse effects of antiretroviral medication resolved after initiating GH replacement. The overlap of unspecific symptoms from long-term medication side effects and GH deficiency can complicate its clinical diagnosis. Importantly, medication adherence was fully restored after initiating GH replacement therapy as the patient underwent a recovery of QoL documented objectively in the routinely performed PROMIS questionnaire.

PROMs are already integral in the assessment of GH replacement therapy initiation, responsiveness, and its side effects, as recommended by clinical practice guidelines (1). Effective feedback of PROMs to the attending physician in clinical routine will facilitate a more individualized approach to treatment and care (8), especially in complex patients with panhypopituitarism. Such patients may face interactions among residual hormone secretion, hormone replacement, and other comorbid diseases and respective treatments with unknown effects on pituitary cell function, such as HIV and antiretroviral therapy (5). Intuitive PROM feedback can support and guide treatment decisions and assess real-world effects of GH replacement and other interventions.

For routine PROM assessment at our center, we employ PROMIS (9). Developed by the National Institute of Health, PROMIS has considerably advanced the field of PROMs using advanced psychometric methods including item response theory, item banking, and computerized adaptive testing. It has been validated in >21,000 members of the US population (9), while the German version has been validated in *n* = >1,500 (6). When implementing this method into the clinical setting beyond valid reference data for the given population, it is important to ensure i) the use of reliable, reproducible, and valid PROMs and an effective presentation of the feedback, and ii) allow for the integration of PROMs and clinical data under careful consideration of data protection concerns, making clinicians’ access to patients’ health data as intuitive, efficient, and comprehensive as possible.

In a complex patient such as ours, where multiple comorbidities and treatments converge, the advantages of utilizing PROMIS to assess QoL become apparent when compared to disease-specific questionnaires. PROMIS enables the documentation of cross-disease effects and the simultaneous consideration of the impacts of several clinical conditions and treatments (9). These effects are often patient-specific rather than confined to a particular disease and might elude detection in a disease-specific questionnaire (10). Optimally, PROM results should not exclusively be compared against cohorts associated with a particular disease but should also be universally comparable against patient groups presenting with more than one medical issue (10).

While we cannot define the exact contribution of GH replacement compared to other factors on the patient’s QoL and adherence to HIV therapy, PROMIS assessments allowed quantification of the QoL improvement after therapy initiation and continued monitoring of possible relevant changes in QoL caused by external factors that might impact clinical decision-making.

In summary, we present how PROMIS can effectively track QoL to enable GH replacement therapy, which can increase medication adherence to HIV therapy. We believe that routine use of PROMs, as well as effective PROM feedback, will help guide treatment decisions in complex cases and facilitate the incorporation of the patient’s voice into routine endocrinological practice.

## Declaration of interest

LHa received speaker fees from Merck and Novo Nordisk and travel grants from Novo Nordisk, Pfizer, and Ipsen. CJS received advisory honoraria or speaker fees from Sandoz, Novo Nordisk, Ascendis, and Pfizer. LM received travel grants from Novo Nordisk, Ipsen, and Pfizer. KM received advisory honoraria or speaker fees from Novo Nordisk, Ipsen, Sanofi, and Recordati. LHe, LR, and JS have no conflicts of interest to declare.

## Funding

This research did not receive any specific grant from any funding agency in the public, commercial, or not-for-profit sector.

## Patient consent

Written informed consent for publication of clinical details was obtained from the patient.

## Author contribution statement

LHe contributed to investigation, visualization, writing the original draft, and writing review and editing. LR contributed to investigation, data curation, and writing review and editing. LM provided clinical care, supervision, and writing review and editing. JS contributed to supervision and writing review and editing. KM contributed to supervision and writing review and editing. CJS provided clinical care, supervision, and writing review and editing. LHa provided clinical care, conceptualization, visualization, supervision, and writing review and editing. All authors approved the final version of the manuscript and agreed to be accountable for all aspects of the work.

## References

[1] Yuen KCJ, Biller BMK, Radovick S, et al. American association of clinical endocrinologists and American College of Endocrinology Guidelines for management of growth hormone deficiency in adults and patients transitioning from pediatric to adult care. Endocr Pract 2019 25 1191–1232. (10.4158/gl-2019-0405) 31760824

[2] Mo D, Blum WF, Rosilio M, et al. Ten-year change in quality of life in adults on growth hormone replacement for growth hormone deficiency: an analysis of the hypopituitary control and complications study. J Clin Endocrinol Metab 2014 99 4581–4588. (10.1210/jc.2014-2892) 25233155

[3] McGauley GA, Cuneo RC, Salomon F, et al. Psychological well-being before and after growth hormone treatment in adults with growth hormone deficiency. Horm Res 2008 33 (Supplement 4) 52–54. (10.1159/000181584) 2245968

[4] van Trigt VR, Pelsma ICM & Biermasz NR. Patient-reported outcomes in refractory hormone-producing pituitary adenomas: an unmet need. Pituitary 2023 26 307–317. (10.1007/s11102-023-01309-4) 37014498 PMC10333395

[5] Rochira V & Guaraldi G. Growth hormone deficiency and human immunodeficiency virus. Best Pract Res Clin Endocrinol Metabol 2017 31 91–111. (10.1016/j.beem.2017.02.006) 28477736

[6] Fischer F, Gibbons C, Coste J, et al. Measurement invariance and general population reference values of the PROMIS profile 29 in the UK, France, and Germany. Qual Life Res 2018 27 999–1014. (10.1007/s11136-018-1785-8) 29350345

[7] Castle-Kirszbaum M, McCormack A, Kam J, et al. Quality of life in non-functioning pituitary adenoma: a systematic review. Neurosurg Rev 2024 47 867. (10.1007/s10143-024-03126-0) 39578273

[8] Gibbons C, Porter I, Gonçalves-Bradley DC, et al. Routine provision of feedback from patient‐reported outcome measurements to healthcare providers and patients in clinical practice. Cochrane Libr 2021 [cited 2025 Mar 24]. (https://www.cochranelibrary.com/cdsr/doi/10.1002/14651858.CD011589.pub2/full) 10.1002/14651858.CD011589.pub2 PMC8509115 34637526

[9] Cella D, Riley W, Stone A, et al. Initial adult health item banks and first wave testing of the patient-reported outcomes measurement information system (PROMIS^TM^) network: 2005–2008. J Clin Epidemiol 2010 63 1179–1194. (10.1016/j.jclinepi.2010.04.011) 20685078 PMC2965562

[10] Cook KF, Jensen SE, Schalet BD, et al. PROMIS® measures of pain, fatigue, negative affect, physical function, and social function demonstrate clinical validity across a range of chronic conditions. J Clin Epidemiol 2016 73 89–102. (10.1016/j.jclinepi.2015.08.038) 26952842 PMC5131708

